# Influence of shift work on the physical work capacity of Tunisian nurses: a cross-sectional study in two university hospitals

**DOI:** 10.11604/pamj.2017.26.59.11279

**Published:** 2017-02-02

**Authors:** Irtyah Merchaoui, Lamia Bouzgarrou, Ahlem Mnasri, Mounir Mghanem, Mohamed Akrout, Jacques Malchaire, Neila Chaari

**Affiliations:** 1 School of Medicine, Occupational Health & Ergonomics Department, University of Monastir, Tunisia; 2 Catholic university of Louvain, Rue rosier bois 75, 1331 Rosières, Belgium

**Keywords:** Nurses, ergonomics, premature aging, shift work, work capacity evaluation, physical tests

## Abstract

**Introduction:**

This study has been performed to determine the influence of rotating shift work on physical working capacity of Tunisian nurses and to design recommendations to managers so that they implement effective preventive measures.

**Methods:**

It is a cross-sectional design using a standardized questionnaire and many physical capacity tests on a representative sample of 1181 nurses and nursing assistants from two university hospital centers of the school of Medicine of Monastir located in the Tunisian Sahel. 293 participants have been recruited by stratified random sampling according to gender and departments. Maximum Grip strength, 30s sit-to-stand test, one leg test, Fingertip-to-Floor test, Saltsa test and peak expiratory flow were used to assess physical capacity. Work ability was assessed through the workability index.

**Results:**

Mental and physical loads were heavily perceived in shift healthcare workers (p=0.01; p=0.02). The maximum grip force was stronger in rotating shift work nurses (p=0.0001). Regarding to the seniority subgroups in each kind of work schedule, the Body Mass Index was increasing with seniority in both schedules. All the physical tests, were better in less-than-ten-year groups. Peak Flow and grip strength were significantly better in less-than-ten-year seniority in shift work group.

**Conclusion:**

There is a need to improve the design of the existing shift systems and to reduce as much as possible shift schedule as well as to avoid shift schedule for over-10-year-seniority nurses.

## Introduction

Rotating shift work is defined as work schedule in which groups of workers rotate through set periods throughout the day [1, 2]. About 20% of European countries workforce and 15.2 million Americans are concerned with this pattern [3]. In developing countries, about 15-30% of the workforce are involved in shift work [2, 3]. In Tunisia, it is estimated that about one fifth of the population is working with a rotating shift. According to Griffiths, the proportion of workers who work in shift schedule is increasing more and more [4]. All over the world, Health care providers are bound to work shift-work to cater for the needs of the sick people and are consequently exposed to the disruption of the synchronous relationship between the body's internal clock and the environment. Many authors have reported that night shift has physical and psychological effects [5, 6]. Night work disrupts the body circadian rhythmicity, sleep alertness and performance [7–9]. It may also have long term health outcomes such as obesity, type 2 diabetes and cardiovascular diseases [7–14]. Night work can also have negative impacts on emotional health, family and social life, drug use and job-related stress. This is particularly damaging for women with family responsibilities (pregnancy and child raising) [3, 6]. For all night workers, and especially nurses, these physical and psychological effects have consequences at the workplace, such as decreased alertness and reduced job performance that could endanger the lives of the patients [15]. The aim of the present study is to determine the influence of rotating shift work on physical working capacity of Tunisian nurses and to design recommendations to managers so that they implement effective preventive measures of premature physical decline to avoid long term health outcomes.

## Methods

**Study design:** A cross-sectional study was conducted to assess the effect of rotating shift work on the physical capacity of nurses.

**Setting:** This study lasted 15 months (from October 2012 to December 2013) and was conducted at the university hospitals of Monastir and Mahdia in Tunisia. Data collection was performed using a questionnaire administered by 4 trained interviewers who did move into different departments to collect data and perform physical tests on nurses.

**Participants:** The sample consisted of 293 nurses, representative of 1181 nurses and nursing assistants. The participants were recruited by stratified random sampling according to gender and departments. All were fully informed of the objectives and the protocol of the study, of the confidentiality of their participation and their data and their right to refuse to participate and to withdraw at any moment. They were requested to sign a written consent to participate.

**Variables:** In our study, rotating shift time work was defined as working in a rotating shift, and day time work was defined as working exclusively during the morning time. The main outcome measures were: *Work ability index (WAI)*: in clinical occupational health and research, this is usually used to assess work ability during health examinations and surveys [16–18]. The first section collects demographic dataand the second one consists of 7 items. WAI is calculated by summing up the points for each item. The total WAI score ranges from 7–49 points, and the scores are categorized into 4 classes: poor (7–27), moderate (28–36), good (37–43) and excellent (44–49). *Perceived physical (PPL) and mental (MPL) workloads:* assessed through a simple question classifying the workload as heavy or light. *Physical (PAD) and domestic (DAD) activity duration:* assessed in hours per week.*The body mass index (BMI):* Expressed in kg/m^2^, it evaluates the degree of obesity and consequently allows the assessment of the associated health risks.

*Physical Capacity*: The physical ability was evaluated by a battery of physical tests: *The Grip Strength Test:* is a measure of the maximum grip force (MGF) using a JAMAR dynamometer (Patterson, Nottinghamshire, United Kingdom). The MGF, expressed in kg, is correlated to the age and the global muscle force of the person [19–21]; *The muscle force of the lower limbs:* assessed through the 30s sit-to-stand test where the number of stands a person can complete in 30 seconds is recorded using a folding chair without arms [22]; *The one leg test (OLT):* consists in measuring the efficiency of postural control on one leg without support on a flat surface. Although many studies have examined the use of the OLT, none has demonstrated conclusively whether it can be used as a practical marker of premature aging or of work capacity evaluation [23]; *Joint flexibility:* is assessed through two types of tests: The thumb- C7 distance and Fingertip-to-Floor test (FTF). The thumb- C7 distance is usually used to determine the degree of flexibility of the shoulder to perform the internal rotation: It measures the distance between the C7 spinous process and the ipsilateral thumb while the hand is behind the same side shoulder [24]. The Fingertip-to-Floor test (FTF) measures the distance between the fingers and the floor when the patient is standing up and trying to touch his toes without bending the knees [25]. These two parameters were measured in centimeters; *Saltsa tests:* are used for screening musculoskeletal disorders (MSDs) of the upper limbs. They can diagnose 12 types of specific MSDs and a general syndrome involving non-specific MSDs but constituting an early indicator of MSDs. These tests were established by the Swedish National Institute for Working Life (NIWL) in 1997 as a research program on working life issues in a European perspective [26, 27]. The total Saltsa score represents the total number of joint disorders in the upper limb; *Pulmonary endurance:* the peak expiratory flow is measured with a peak flow meter which is a handy, easy-to-use tool to assess air flow limitation. It can be an important aid in both the diagnosis and monitoring of asthma. The literature has reported that this flow is correlated to gender, height and age. In fact, during ageing, there is a progressive decline in respiratory function, because of a loss of respiratory muscle strength, increased stiffness of the chest wall and reduced elastic recoil of the lung and diffusion capacity [28]. In our study we used the rate Z pick flow defined as the pick flow divided by the height, to assess pulmonary endurance.

**Data measurement:** The first approach was to compare the two groups’ variables of daytime and rotating shift time nurses and give a physical assessment. The second approach was to divide the groups of shift schedule and day time nurses according to job seniority to find out differences within these sub-groups.

**Statistical methods:** The data were analyzed using the SPSS 21 software. Descriptive statistics were used to describe the population characteristics by calculating the frequencies and the percentages for categorical variables on the one hand, and the means, standard deviations and extent of extreme values for quantitative variables on the other hand. The chi-square test was used to check whether the associations found presented a statistical significance (p< 0.05). The Student t-test was used to compare group means. The cross-tabulation analysis was used to analyze categorical data.

## Results

### First step: according to work schedules

Our survey sample of 293 was representative of 1181 nurses and nursing assistants with 111 day time nurses, and 182 rotating shift time working nurses. Sex ratio was about 1.061. In our sample, as shown in Table 1, the mean age was about 44.26 years for the participating nurses working in daytime schedule and about 41.65 years for those working in shift time schedule. The analysis of socio-demographic factors revealed that job seniority was associated with work schedules (p=0.026). Actually, nurses working in day time schedule seem to be more experienced than those of rotating shift schedule (mean range for job seniority equal to 20.62 years and 17.12 years respectively). The perceived physical and mental workloads were significantly associated with work schedules (p=0.02 and 0.01 respectively) (Table 1). The mean WAI score was about 40 ± 6.28. According to the WAI categorical classification, most of the participants had a good or excellent work capacity (75%). The independent-sample t showed no significant association between work schedule and work capacity (p >0.05) (Table 1). Concerning the physical capacity tests, the Grip strength test (p=0.001) and the pulmonary endurance (p=0.05) were the only physical tests to show an association with the rhythm of work. In fact, nurses working in shift times seemed to have better muscle force and better pulmonary endurance than those working in day time schedule (Table 1).

**Table 1:** Socio-professional characteristics, physical and cognitive tests of the sample according to work schedule

Variables	Day time group		Shift work group		p
	N=111	Mean± SD	N=182	Mean± SD	
**Socioprofessional Characteristics**					
Age (years)		43.84±12.56		41.65±11.63	0.12
Seniority (years)		20.62±13.13		17.12±12.9	***0.02***
WAI		39.72±6.24		40.45±6.28	0.11
PAD (hours/W)		2.59±5.14		3.62±6.54	0.48
DAD (hours/W)		14.40±16.57		14.86±15	0.80
**PPL**					
slight	16		15		***0.02***
heavy	95		167		
**PML**					
Slight	13		12		***0.01***
heavy	98		170		
BMI (Kg/m^2^)		26.27±3.9		26.63±3.49	0.44
Grip Strength Test (kg)		47.18±21.93		57.95±1.66	***<0.001***
Lower Limb muscle force		19.04±6.53		18.11±5.45	0.018
One leg test (seconds)		26.54±5.09		27.35±5.03	0.018
The thumb-C7 distance		6.95±9.04		7.38±7.4	0.65
Finger to floor test (cm)		16.04±6.4		16.65±5.59	0.39
Saltsa test		2.83±0.26		2.56±0.31	0.34
Z Peak Flow (l/mn.cm)		0.70±0.015		0.73±0.13	0.05

**Second step: according to Job seniority within each schedule group:** The comparison between nurses within the shift schedule group based on job seniority showed that over-ten-years of seniority nurses in both schedule groups were more likely to have weight gain (p= 0.001). The workability index was significantly decreased with job seniority within the shift schedule work group (p=0.01) (**Table 2**). Otherwise, Peak flow test (p=0,014), salsa test, one leg test (p=0.001), muscle force of lower limb test (p=0.001) finger to floor test (p=0.001) and the thumb-C7 distance (p=0.045) were significantly better within the group with less than 10 years job seniority (Table 2). Concerning the influence of job seniority on different variables within the day time group, it seems that the over-ten-year group in day time work had a significantly more time to perform physical activity (p=0.034). they were also more likely to have weight gain (p=0.001). T-tests were used to analyse the relationship between seniority within the day time group and the different capacity physical tests and showed that the lower limb test (p=0.0001), the one leg test (p=0.003), the thumb-C7 test (p=0.001), finger to floor test (p=0.022) and Saltsa test (p=0.0001) were better in the less-than-ten-year group in the day time group (Table 2).

**Table 2:** Socio-professional characteristics and physical tests of the sample according to job seniority within each schedule group

Variables	Job seniority in shift work group				p	Job seniority in day time group				p
	<10 years		≥10 years			<10 years		≥10 years		
	N=88	Mean±SD	N=94	Mean±SD		N =37	Mean±SD	N	Mean±SD	
**Sociodemographic Characteristics**									
Age (years)		30.72	51.89	27,88	0.0001		29.65		50.93	0.0001
WAI		41.65		39.31	0.01		40.05		38.87	0.35
PAD (hours/W)		2.66		4.53	0.05		1.14		3.32	0.034
DAD (hours/W)		17.23		12.65	0.04		11.51		15.84	0.19
**PPL**										
slight	8		7		0,07					
heavy	80		87							
**PML**										
Slight	7		5							
heavy	81		89		0,05					
**Physical Tests**										
BMI (Kg/m^2^)		25.3		27.88	*0.001*		24.5±2.9		27.1±4	***0.001***
Grip Strength Test (kg)		57.2±23.3		58.6±21.6	0.6		51.6±22.8		44.9±21.2	0.127
Lower Limb muscle force		19.8±5.3		16.5±5.04	*0.001*		22.0±5.3		17.5±6.5	***0.0001***
One leg test (seconds)		29.1±3.0		25.4±5.6	*0.001*		28.6±4.2		25.3±5.8	***0.003***
The thumb-C7 distance		15.8±4.8		17.4±6.1	*0.045*		13.35±5.9		17.4±6.4	***0.001***
Finger to floor test (cm)		4.8±6.7		9.7±7.2	*0.001*		4.1±7.5		8.3±9.4	***0.022***
Saltsa test		0.46±1.6		2.0±3.11	*0.001*		0.18±0.5		2.3±3.2	***0.0001***
Z Peak Flow (l/mn.cm)		444±89		412±83	*0.014*		428±95		396±94	0.103

## Discussion

The purpose of our study was to examine if and how work ability and physical capacities were associated with rotating shift work among Tunisian nurses. Taken together, the study findings suggest that mental and physical loads were heavily perceived in shift healthcare workers (p=0.01; p=0.02) and that the maximum grip force was the only physical variable significantly influenced by the rhythm of work. In fact, maximum grip force was stronger in rotating shift work nurses (p=0.0001). These were the only effects of rotating shift work on nurses’ physical capacity of work. As for the question of seniority subgroups in each kind of work schedule, the Body Mass Index increased with seniority in both schedules. All the physical tests, except the grip strength test and the Peak Flow, were better in less-than-ten-year groups in shift and day time schedules. Finally, Peak Flow was significantly better in less-than-ten-year seniority in the shift work group. Mental and physical workloads were significantly perceived by shift-schedule nurses. The events giving rise to such a perception are probably in relationship with the lack of staff during shift work whereas shift work nurses have exactly the same work conditions and strain as day time nurses.

These results are in accordance withthose observed in earlier studies which concluded that inadequate and atypical schedules may impact health [29–31]. This may result in a reduced quantity and quality of sleep, a decline in cognitive and physical performance and an associated increased risk for errors and accidents and interference with the nurse's family and social commitments [31]. Moreover, the disruption of circadian rhythm and the shift work conditions (such as lightening, temperature and the reduction of in the number of available workforce and services) influence the perception of one's work capacity [29]. All these negative consequences may be perceived by a worker as a mental and physical job strain reducing his capacity to perform his work. Contrary to expectations, this study did not prove any significant association between work schedules and work ability index.

These findings corroborate those of CAMERINO stating that Work schedule is not related to changes in WAI [32]. These findings could be explained by the “napping phenomenon”. In fact, while performing data collection, we noticed that most of the rotating shift nurses had a nap during shifts and shared the work tasks together. Besides, a fixed work shift team is a helpful concept to cope with shift work because relationships between coworkers would be stronger and it would be easier to communicate and understand each other and to perform better work tasks. In the literature, napping is even recommended in some studies to help nurses cope with shift work and to make it less stressful [33]. For night shift nursing, Da SILVA found that napping during the 12-hour night shift results in less sleepiness at work and less need for recovery after work [34]. But, despite the absence of association between the WAI and work schedule in our study, we could prove that a less than 10 years job seniority was a predictor of a better work ability index within the shift schedule group (p=0.01). This finding corroborates the shift-schedule-seniority effects and consequently aging effects on workability index, agreeing with the findings of other studies [16, 35].

It is obvious that work capacity is a dynamic process that changes greatly according to several reasons, apart from ageing, throughout an individual's work life. In fact, it is well known that shift work could interfere with the circadian sleep/wake cycle resulting in excessive sleepiness and fatigue interfering with performance and health [15]. Previous cross sectional studies specifically related to nursing profession and health care personnel showed that work capacity as assessed by the Work Ability Index (WAI) decreased with age in shift schedules and found a significant association between sleep problems and WAI [36]. No significant differences were found in muscle performance tests apart from the grip strength test which was the unique muscular physical capacity test to be significantly improved by rotating shift work (p=0.0001). The versatility of tasks requiring hand force and dexterity, while there is added to a lack of staff during shifts, might be a possible explanation for this [37]. However, this result has not previously been described. BAMBAEICHI, did not bring any proof of the influence of time of the day and partial sleep deprivation on muscle strength in eumenorrheic females [38]. Furthermore, grip strength has been used as a predictor of functional limitations for upper body limbs and even as all-cause mortality predictor in healthy population [39, 40].

Concerning joint flexibility, the Chi-square test did not show any significant differences between shift time and day time schedules in the thumb-C7 distance test or the finger to floor test. However, these tests seem to worsen according to seniority in shift work (p=0.001 / 0.001) or day time work (p=0.001/0.0001). This finding suggests that the breakdown of joint flexibility in our population is due to seniority at work and thus to age and not to shift schedules. This is consistent with earlier observations which showed that age-related changes that occur in the cartilage matrix can contribute to the decrease of joint flexibility in elderly and to the development of osteoarthritis [41, 42]. Moreover we couldn’t prove any statistical link between rotating shift schedule and Saltsa test. However, this test was significantly linked to seniority within day time work (p=0.0001) group and shift time work (p=0.001) group. Consequently, Saltsa tests regrouping all the physical tests screening for musculoskeletal joint disorders in the upper limb had a significant relationship with seniority and thus were age-related. In the literature, nurses working in shift schedule reported especially low back and neck pain compared to daytime nurses who reported upper extremities pain and were at great risk of sustaining an occupational musculoskeletal injury [43, 44]. The risk of low back pain could be explained by the highly physically demanding postures and tasks required in shift schedule such as patient transfers. The long exposure to this physical postural risk could lead to MSD among shift nurses [44]. Interestingly, no reference was made to seniority in the literature for the physical tests while cognitive decline is well documented.

Concerning pulmonary performance, there was no significant relation between its index and nurses’ work schedule. However, our findings suggest that an over 10- year - exposure to shift work within the shift work group affects pulmonary performance. This could be related to aging because of the significance of age distribution between both groups. The concept of lung physiological aging has been well documented in the literature [16]. Several limitations are to be considered with respect to this study: in order to avoid any linguistic misunderstanding, 4 trained interviewers did explain questionnaires in Tunisian dialect. Moreover, the study was a cross-sectional one in design but a longitudinal study would be more appropriate to achieve higher levels of evidence of nurses' work capacity progression during their professional career. Moreover, given the length of the questionnaire and the tests and the lack of time for some nurses, some of responses were qualified as rushed (especially for nurses of the emergency and intensive care units) and we had to set up further appointments to perform the survey as requested.

## Conclusion

The findings of our study have a number of practical implications. In fact, the recommendations should be focused on implementing effective preventive measures in hospitals to encourage health promotion of nurses and other healthcare workers. Most studies rely heavily on the general scientific literature in the field of shift work assuming that it is associated with health problems. Since there is no ideal shift, the focus of the literature has been on improving the design of the existing shift systems and on reducing as much as possible shift schedule and quick change from night to day work [45–48]. This suggests that hospital managers and Heads of Departments should be aware of the necessity of avoiding shift schedule for over-10-year-seniority and giving nurses the freedom of choice regarding shifts [30]. This can be one possible implication of our results regarding the fact that work capacity will be worse after 10 years of seniority. Consequently, it would be better to automatically change nurses from shift work to day time work starting from this limit. On the other hand, recovery time and rest breaks or naps must also be considered, supported and legally implemented. This issue is widely cited in the literature. In fact, effective napping strategies are highly required such as planning and ensuring shift nurses take a rest or 20 minute-naps, creating a nap room that is quiet, safe, clean and close to units, including timers to alert nurses at the end of the nap period [45, 47]. Personal measures are also required such as physical exercise, healthy and balanced meals and avoiding smoking. Nurses of both schedules should be aware of these, and occupational health physicians have many opportunities to emphasize on these personnel-related measures in every occupational check-up. This may reduce the negative impact of shift work and improve tolerance to this work pattern. Occupational health may also reduce nurse fatigue by developing a fatigue risk management program including Primary, Secondary and Tertiary interventions. The literature also highlights some techniques and therapies used to deal with the circadian disturbance such as bright light and blue light which have not been yet experimented in our hospitals [48]. Taken together, these results suggest that there is a need for further clarification of the consequences of the rotating shift system in the clinical environment and the adoption of a shift system with fewer physical health implications through further longitudinal studies.

### What is known about this topic

- Cognitive decline due to shift work in nurses is well documented;
- No reference in the literature is made to seniority to assess physical capacity through physical tests.

### What this study adds

- Physical ability tests could be useful to assess the physical capacity at work;
- Physical tests have proved that physical capacity at work is declining with seniority in shift work.
